# Understanding parental involvement in the Positive Behavioral Interventions and Supports (PBIS) process in schools: a qualitative study

**DOI:** 10.3389/fpsyg.2026.1802372

**Published:** 2026-05-08

**Authors:** Siddiq Ahmed, Morgan Chitiyo, Mohammed Al Jaffal, Hamad Hamdi

**Affiliations:** 1 Department of Teaching and Learning, American College of Education, Indianapolis, IN, United States; 2 Department of Specialized Education Services, University of North Carolina Greensboro, Greensboro, NC, United States; 3 Department of Special Education, College of Education, King Saud University, Riyadh, Saudi Arabia

**Keywords:** Behavior Intervention Plans, functional behavior assessments, individualized educational plan, parental involvement, Positive Behavior Support

## Abstract

**Introduction:**

Students with emotional and behavioral disorders (EBD) often experience difficulties in academic and social functioning. Positive Behavioral Interventions and Supports (PBIS) is widely used to address these challenges; however, parents’ experiences and involvement in PBIS remain underexplored.

**Methods:**

This study employed a qualitative design using Interpretative Phenomenological Analysis (IPA). Semi-structured interviews were conducted with eight parents of children receiving PBIS support. Data were analyzed through iterative transcript review, identification of emergent themes, and cross-case analysis.

**Results:**

Findings revealed that parents had limited understanding of PBIS and reported minimal involvement in decision-making processes. Key barriers included communication gaps, unclear expectations, and limited structured collaboration. Although parents expressed willingness to support their children, many felt excluded from the PBIS process.

**Discussion:**

The findings emphasize the need to strengthen family–school communication and create more inclusive opportunities for parental engagement. Enhancing shared decision-making and clarifying parental roles may improve PBIS implementation and support better outcomes for students with EBD. Implicatons for practice and future research are discussed.

## 1 Introduction

Positive Behavioral Interventions and Supports (PBIS) is a systematic approach that is designed to establish a positive school culture across school settings (Horner et al., 2009). Turnbull et al. (2002) defined PBIS as a behaviorally based systems approach used to strengthen the capacity of schools, families, and communities to create effective environments that improve the alignment between evidence-based practices and the settings where teaching and learning take place (p. 59). The purpose of PBIS is to promote academic and social success for all students, particularly those with challenging behavior (Lloyd et al., 2023). Thus, PBIS aims to enhance an individual’s quality of life by promoting greater independence through adjustments to their environment (Gage et al., 2018).

PBIS is a three-level system. The first level (Tier 1) includes school-wide rules and supports designed to prevent behavior problems and create a positive environment for all students and staff. The second level (Tier 2) provides extra support for students who do not respond to the first level, focusing on reducing behavior issues in specific settings like classrooms, hallways, or cafeterias. The third level (Tier 3) offers intensive, individualized support for students with serious behavioral challenges who need more targeted interventions beyond the first two levels. At this level, functional behavior assessments (FBA) are conducted, and individualized behavior intervention plans are developed in order to provide students with individualized support (Malloy et al., 2018). In order to maximize the effectiveness of PBIS, it is important to involve families in the PBIS process (Strickland–Cohen et al., 2021). A few studies (e.g., Blair et al., 2011; Hewitt et al., 2016; Strickland–Cohen et al., 2021) demonstrate the benefits of family involvement in implementing PBIS.

Furthermore, research demonstrates the importance of the family in the school-wide Positive Behavior Support (SWPBS) process in increasing students’ quality of life and learning (Walker et al., 2023). Dunlap and colleagues stated that Families are considered experts regarding their children their children. Professionals cannot effectively support a child without meaningful family contribution to goal development and planning to define and develop goals for a behavior support plan (Dunlap et al., 2001). When parents are involved with school systems in PBIS interventions, it promotes shared responsibility, joint decision-making, and more effective communication and information sharing between families and schools. This partnership promotes the effectiveness and sustainability of PBIS outcomes (Lucyshyn et al., 2007; Strickland–Cohen et al., 2021).

Although family involvement in PBIS is important, the level of participation differs greatly across schools, ranging from full engagement to no involvement at all. Family involvement is emphasized as essential for setting goals and creating guidelines for children with disabilities to support positive long-term outcomes. It is also argued that placing families at the center of the PBIS process helps to create practical and contextual goals for individuals with challenging behavior (Rose et al., 2024; Lucyshyn et al., 2007). Since the main goal of PBIS is to improve the quality of life for both the individuals with challenging behavior and their families, the partnership between families and professionals is extremely important in implementing SWPBS (Walker et al., 2023). Although previous research has emphasized the importance of family involvement in PBIS implementation, much of the existing literature focuses on program effectiveness and system-level outcomes. Limited attention has been given to how parents themselves experience and interpret their involvement in the PBIS process. Understanding parents’ perspectives provide deeper insight into how collaboration is perceived and operationalized in practice. More specifically, this study focuses on parental involvement at the Tier 3 level of PBIS. At this level, students receive individualized support such as (BIPs) and (FBAs). This focus helps provide a clearer understanding of parents’ experiences in more intensive intervention settings.

## 2 Research questions

1. How do parents involved in the PBIS process perceive and describe their experience supporting children with challenging behaviors?

2. How do parents understand and make sense of their participation in school-based PBIS interventions for children with challenging behaviors?

## 3 Methodology

### 3.1 Research design

This study used a qualitative approach based on Interpretative Phenomenological Analysis (IPA) to explore how parents understand and experience their involvement in the Positive Behavioral Interventions and Supports (PBIS) process in school settings. IPA is grounded in phenomenology and focuses on understanding how individuals make sense of important experiences in their lives (Smith et al., 2021). In this research, the main attention was given to how parents perceived their roles, their communication with schools, and their level of participation in PBIS implementation. The purpose of the study was to gain a deeper understanding of parents’ personal experiences and perspectives. This approach helped to examine closely the different personal and contextual factors that influence their involvement.

Semi-structured interviews were conducted by the first author with eight parents whose children had received PBIS interventions in their schools. The small and purposively selected sample is consistent with IPA methodology, which emphasizes detailed and in-depth analysis of participants’ experiences instead of large sample size. During the research process, reflexivity was considered important to ensure credibility. The researchers reflected on their own professional experiences and possible assumptions related to PBIS and parental involvement. By recognizing these positions, efforts were made to present participants’ views as accurately and respectfully as possible.

### 3.2 Participants

Eight parents who had firsthand experience implementing School-Wide Positive Behavioral Interventions and Supports (SWPBIS) in their child’s school and whose children had been diagnosed with Emotional and Behavioral Disorders (EBD) participated in this study. Purposive sampling was used to choose participants in accordance with the principles of Interpretative Phenomenological Analysis (IPA) in order to guarantee depth, relevance, and experiential insight rather than representativeness or generalizability. Participants were chosen from Maryland and Pennsylvania public schools that had been using SWPBIS for at least 3 years. The goal of concentrating on schools with long-term PBIS implementation was to make sure parents were exposed to the structures and procedures that were being studied.

Participants had to meet the following requirements in order to be eligible: (a) be a parent or legal guardian of a child who has been identified as having challenging behavior and is receiving special education services; (b) have a child with an active Behavior Intervention Plan (BIP) receiving Tier 3 PBIS support; (c) have at least 2 years of experience working with school staff on behavioral planning or intervention; (d) have a child enrolled in kindergarten through eighth grade; (e) be associated with a school that has used PBIS for at least 3 years; and (f) agree to participate in an audio-recorded, semi-structured interview. Every participating parent has been in constant communication with school personnel regarding PBIS-related interventions for more than 2 years. A variety of ethnic backgrounds were represented in the sample: three participants identified as African American, three as Arab, one as Asian, and one as White American. Table 1 provides a comprehensive overview of participant characteristics.

**TABLE 1:** Demographic characteristics of participants.

Characteristic	Description
Number of Participants	8 parents
parent role	Parents/guardians of children with EBD
Ethnicity	1 White American; 1 Asian; 3 African American; 3 Arab
Geographic location	Schools located in Pennsylvania and Maryland
Child grade range	Kindergarten through Grade 8
PBIS experience	Minimum of 2 years of collaboration with school
Child support level	All children had Behavior Intervention Plans (BIPs) and received Tier 3 PBIS support
Child grade range	8 parents

### 3.3 Ethics statement

The Institutional Review Board (IRB) at Duquesne University approved this study. Participants were informed of the study’s purpose, the voluntary nature of participation, and their right to withdraw at any time. Pseudonyms were used to protect confidentiality, and identifying information was removed from reports and transcripts.

### 3.4 Data collection

Data were gathered using semi-structured interviews, and the interview protocol was designed based on a review of relevant literature on PBIS and parental involvement. The questions explored parents’ understanding of PBIS, their experiences collaborating with schools, their perceived level of involvement, and their interest in greater participation. Semi-structured interviews were selected because they allow participants to describe their experiences in their own words while still providing structure to guide the discussion. This method also enables the researcher to ask follow-up questions when clarification is needed, ensuring that differences in responses reflect participants’ perspectives rather than variations in questioning (Fox, 2009). Each interview was audio-recorded with participants’ consent and later transcribed verbatim for analysis.

### 3.5 Data analysis

This study used the fundamental steps of Interpretative Phenomenological Analysis (IPA) to analyze the data. Each interview transcript was read multiple times to fully comprehend the individuals’ lived experiences and to become intimately acquainted with the material. Immersion in the viewpoints of the participants was facilitated by this recurrent interaction with the data. Detailed notes were taken directly on the transcripts throughout the subsequent phase. These annotations contained linguistic observations about the participants’ language use, descriptive remarks about their accounts, and preliminary interpretive thoughts. Emergent themes were established for each case based on these notes. Each transcript’s connections between topics were then analyzed to group them into more comprehensive conceptual categories. Themes were compared among participants once each case’s analysis was finished in order to find both commonalities and differences in parental experiences. Higher-order themes that honored individual distinctions while capturing common patterns were developed as a result of this process. The researcher kept reflective notes during the analysis process to record interpretive choices and improve the findings’ transparency and reliability.

## 4 Results

The parents’ interviews resulted in the identification of two clusters and nine key themes. Each theme is introduced first, followed by the relevant responses from parents. Participant numbers and pseudonyms for the children and schools were assigned accordingly.

### 4.1 Cluster 1: parents’ participation experience in the PBIS interventions

Four themes emerged from this cluster explaining parents’ prior knowledge and understanding of PBIS (see Table 2): (a) knowledge and understanding of PBIS, (b) views on PBIS implementation, (c) how parents learned about PBIS, and (d) satisfaction with PBIS. Parents reported gaining information about PBIS through working with school systems, college studies, and school-provided training or information sessions. All eight parents showed some level of understanding by describing PBIS and how it was implemented at home.

**TABLE 2:** Prior knowledge and expectations regarding Positive Behavioral Interventions and Supports (PBIS).

Themes	Parent participants							
	1	2	3	4	5	6	7	8
Parents knowledge and understanding of PBIS	X	X	X	X	X	–	X	X
Parents’ views about PBIS implementation	X	X	X	X	–	–	–	X
How parents learned about PBIS	X	X	X	X	X	–	X	X
Parent’s satisfaction with PBIS	X	X	–	X	–	X	–	–

#### 4.1.1 Parents’ knowledge and understanding of PBIS

This theme reflects parents’ existing knowledge and understanding of PBIS. All parents reported being familiar with PBIS concepts prior to implementation, including its principles, behavior management systems, use of positive reinforcement and rewards, reduction of challenging behaviors, and teaching appropriate alternatives. They recognized that PBIS involves clear rules, structured guidance, and consequences for inappropriate behavior.

Parent 1 described PBIS as “a research-based system to manage behavior through clear rules, guidance, and consequences.” Parent 2 noted that it “helps address behavioral issues, improve focus, and support better academic outcomes.”

This theme shows that parents, particularly Parent 3, viewed Tier 3 PBIS as targeted support for children with challenging behaviors or special needs. They understood it as a system that helps redirect negative behaviors into positive ones through guidance, reinforcement, and incentives, ultimately improving behavior and learning outcomes.

Parent 3 described it as “support that helps challenging students manage and redirect behavior in positive ways.”

These responses suggest that parents understood PBIS primarily as a structured behavioral management system. However, their explanations focused more on observable procedures than on underlying processes such as FBA or individualized planning. This may indicate that parents interpret PBIS mainly through visible practices rather than through its conceptual framework.

#### 4.1.2 Parents’ views about PBIS implementation

This theme highlights parental involvement in PBIS implementation at both school and home. Parents reported positive experiences and saw PBIS as effective in reducing challenging behaviors and teaching appropriate ones. Their collaboration with schools and use of provided strategies at home helped improve their children’s behavior, although consistency between school and home was sometimes limited.

Parent 1 said it is “a positive approach that solves many behavior issues.” Parent 2 noted it “played a big role in his progress.” Parent 3 observed it “works at school but not always at home.”

The parents’ responses indicated that they viewed their engagement with the school as beneficial due to the accurate instruction and direction that they received from the schools to apply in the home environment. They also highlighted that their engagement with the school as a partner played a vital role to change their children’s behavior. Overall, they found PBIS to be a pleasant experience for them and their children, which promoted positive learning. Finally, the participants noted that the implementation of PBIS in the school is crucial in helping their child to acquire appropriate behavior.

Based on the parents’ descriptions, there was a high connection between their assessment of PBIS and their children’s observable behavioral change. Parental satisfaction may be more dependent on observable change than on involvement in decision-making processes, since their evaluations of success were based on tangible results rather than procedural comprehension.

#### 4.1.3 How parents learned about PBIS?

Parents learned about PBIS mainly through schools, especially via IEP/BIP plans and communication with teachers or principals, while some also learned through workshops, personal study, or specialists. Parent eight stated: “I learned about this from the teachers; I took psychology too back home I learned a bit about it as well.” Parent 1 said that: “I learned through workshops.”

The different pathways through which parents learned about PBIS indicate variability in informational access. Parents who were educators or had professional exposure appeared more confident in describing the framework, which may reflect how background knowledge shapes parents’ sense of involvement and influence within the process.

#### 4.1.4 Parent’s satisfaction with PBIS

Most parents were satisfied with PBIS, viewing it as helpful and effective in improving behavior, Parent 2 explain that the PBIS was very positive and effective program, though one parent was dissatisfied due to poor communication and limited focus on behavior. Parent 5 mentioned the school did not clearly explain PBIS and focused more on academics than behavior. Although most parents expressed satisfaction, this satisfaction appeared to be closely linked to improvements in their child’s behavior rather than to the quality of collaboration itself. This suggests that parental approval of PBIS may coexist with limited participation in its planning processes.

### 4.2 Cluster 2: parental efforts in the implementation of PBIS

The second cluster revealed five themes related to how parents contributed to the implementation of PBIS (a) Parents’ motivation to be involved in PBIS with the school system (b) Parents’ contribution and support in developing BIPs (c) Parents’ roles and support to schools in implementing PBIS (d) Parents’ involvement in FBA (e) Communication regarding the child’s behavior. These themes are presented in Table 3.

**TABLE 3:** Parental efforts in the implementation of Positive Behavioral Interventions and Supports (PBIS).

Themes	Parent participants							
	1	2	3	4	5	6	7	8
Parental motivation to be involved in PBIS with the schools	X	X	X	X	X		X	X
Parents’ contribution and support in developing BIPs	–	X	X	X	X	X	X	X
Parents’ roles and support to schools in implementing PBIS	X	X	X	X	X	X	X	X
Parents’ involvement in FBA	–	–	X	X	X	X	X	X
Communication regarding the child’s behavior	X	X	X	X	X	X	X	X

#### 4.2.1 Parental motivation to be involved in PBIS with the schools

This theme describes parents’ strong motivation to participate in PBIS and collaborate with schools in implementing their child’s BIPs. Most parents viewed themselves as equal partners with the school and emphasized shared responsibility in managing and improving behavior. They reported involvement through regular communication, applying strategies at home, and attending IEP meetings. Despite some communication challenges for one parent, overall parents valued their active role in supporting behavioral change. Parent 1 stated, “Parents play a big role with the school, 50/50, and we work together at home.” Parent 2 stated, “It was family-based so everyone stayed on the same page and worked together.”

Parents’ emphasis on shared responsibility reflects their desire for equal partnership. Their accounts suggest that motivation was not lacking; rather, involvement depended on how collaboration structures were organized within the school context.

#### 4.2.2 Parents’ contribution and support in developing BIPs

This theme explains how parents supported schools in developing and implementing BIPs by reinforcing the same strategies at home. Most parents reported closely collaborating with teachers and maintaining daily communication to ensure consistency between school and home interventions. Parent 2 described coordinating with the school and replicating the school routine at home, even during home services. Parent 3 stated, “When he received in-home services, they implemented the behavior plan and we continued it in our daily routine after they left.”

Parents’ descriptions of how they reinforced Behavior Intervention Plans (BIPs) at home demonstrate their commitment to maintaining consistency across settings. However, their role appeared to be primarily operational rather than strategic, as their involvement was more often limited to implementation of plans rather than participation in decision-making processes.

#### 4.2.3 Parents’ roles and support to schools in implementing PBIS

This theme describes the specific roles parents played in supporting PBIS implementation at home. All parents reported reinforcing school strategies by working with their children on BIPs, teaching expected behaviors, using rewards and consequences, and maintaining daily routines. They also shared responsibility with schools by following PBIS guidelines and communicating any concerns or progress. Parent 4 added that parents also shared useful information with teachers to support effective intervention. Overall, parents emphasized consistent collaboration and support for behavior improvement.

Parent 1 stated, “We talk with the child, use encouragement and consequences, and reinforce rules and rewards at home.” Parent 2 stated, “I support by staying consistent with their goals, teaching skills, and keeping in touch to ensure progress.” Parent 3 stated, “I kept in contact, shared updates, and worked with them by providing input and needed support.”

These findings suggest that parents viewed themselves as actively supporting PBIS practices within the home environment; however, their involvement appeared to be largely shaped by expectations established by the school. This pattern may point to a disparity between parents’ efforts to contribute and the level of authority retained by the institution.

#### 4.2.4 Parents’ involvement in FBA

This theme explains how parents contributed to conducting FBAs by sharing important information to help schools understand their child’s behavior. Most parents reported active involvement through daily communication, describing their child’s behaviors, triggers, and responses, and collaborating with teachers to support behavior assessment and planning.

Parent 3 stated, “I kept them updated about his behavior and worked with them on concerns, and they guided me on how to respond at home.” Parent 4 stated, “I provided information about my child’s behavior at home and school, including triggers, and we used a communication notebook to share updates.”

Although parents indicated that they contributed information during Functional Behavioral Assessments (FBAs), their involvement seemed to function more as consultation than true collaboration. This implies that their participation was primarily confined to sharing information rather than engaging in joint analysis or shared decision-making processes.

#### 4.2.5 Communication regarding the child’s behavior

This theme describes how parents communicated with schools about their children’s behavior. Most parents reported frequent, often daily communication using emails, text messages, communication notebooks, and scheduled school meetings to discuss behavior and progress. Parent 1 explained that contact mainly occurred during school meetings a few times a year and only when issues arose. Parent 2 reported daily email communication with teachers who regularly updated her on her child’s progress. Parent 3 noted that communication was frequent in earlier school years but decreased over time, while another parent expressed concern about limited communication and lack of updates regarding the behavior plan.

Shared ownership of intervention planning did not always follow from regular communication. Although parents reported frequent communication, there were differences in the depth and reciprocity of that communication, which could affect how partnership is perceived.

## 5 Discussion

This study explored how parents of children with emotional and behavioral disorders (EBD) experienced their participation in the PBIS process. The findings indicated that parental involvement was shaped by how parents understood their roles within the educational system, as well as by their access to formal procedures. Participants’ accounts suggested that engagement in PBIS is influenced by both school policies and the quality of relationships established with school personnel. The findings also reflected parental involvement at Tier 3 of PBIS, where involvement is typically more intensive and focused on individualized plans. In contrast, involvement at Tiers 1 and 2 is often less intensive. This difference may explain why parents in this study were more involved in supporting interventions at home than in decision making.

### 5.1 Parental knowledge of PBIS

Parents in this study reported having a limited understanding of PBIS procedures, even in cases where schools indicated that explanations had been provided. Although some participants were able to describe general concepts, many demonstrated uncertainty regarding specific elements, such as the Functional Behavioral Assessment (FBA). These findings suggest that simply providing information does not automatically lead to meaningful understanding.

This limited comprehension appeared to shape how parents viewed their capacity to contribute. When parents did not clearly understand the PBIS framework, they expressed hesitation about how to reinforce or support its implementation at home. Prior research has highlighted the importance of equipping parents with adequate knowledge of behavioral support practices to ensure consistency across home and school environments (Hieneman and Fefer, 2017). The present findings build on this perspective by suggesting that knowledge also influences parents’ sense of legitimacy and confidence in their involvement.

Likewise, insufficient understanding of behavioral frameworks may weaken intended outcomes when parents are uncertain about how to align home-based practices with school expectations (Minke and Anderson, 2005). Importantly, the uncertainty expressed by parents in this study was not framed as resistance, but rather as a lack of clarity that influenced the depth of their engagement.

### 5.2 Parental role in PBIS

Participants described their roles as constrained and largely reactive. Their involvement primarily consisted of responding to school-initiated communication and reporting behavioral changes observed at home. Only a small number of parents indicated that they were actively engaged in planning discussions. This perceived imbalance appeared to shape their understanding of collaboration within the PBIS framework.

Effective collaboration requires reciprocity and shared responsibility between families and professionals (Dunlap and Fox, 2007). However, parents in this study frequently characterized communication as one-directional. Although schools maintained regular contact, parents did not consistently experience inclusion in decision-making processes.

Prior research suggests that meaningful parental involvement enhances self-determination and contributes to improved behavioral outcomes (Hieneman and Fefer, 2017; Mumbardó-Adam et al., 2023). The present findings align with this perspective while also revealing a gap between parents’ willingness to engage and the structural opportunities available to them. Participants expressed readiness to contribute but described limited guidance regarding how to participate in more substantive ways.

Similarly, previous scholarship links parental involvement to positive academic performance and behavioral development (Hart, 2011; Chu, 2015). The experiences reported in this study extend this understanding by suggesting that involvement entails more than presence; it requires shared ownership in intervention planning. Supporting families in implementing PBIS practices at home has been proposed as a strategy to enhance consistency across settings (Garbacz et al., 2018), and the present findings underscore the importance of such alignment.

### 5.3 Parents’ motivation to be involved in PBIS with school systems

One important finding of this study is that parents expressed a clear desire to be more actively involved in PBIS. Rather than conveying frustration or disengagement, they repeatedly indicated an interest in gaining deeper understanding and playing a more meaningful role in the process.

Earlier research has identified parents as essential contributors to effective PBIS implementation (Blair et al., 2011; Dunlap et al., 2001). The findings of the present study suggest that limited engagement may not stem from insufficient parental commitment, but instead from the way participatory structures are organized within schools. Parents portrayed themselves as ready to engage, yet positioned at the margins of decision-making.

Previous scholarship has also demonstrated that collaboration with families supports the identification of student strengths and needs while contributing to improved behavioral outcomes (Bailey and Blair, 2015; Blair et al., 2011). In the current study, parents viewed themselves as highly familiar with their children’s behavioral patterns, but uncertain about how their knowledge was integrated into formal intervention planning.

Although earlier studies have pointed to difficulties in parent-school collaboration as obstacles to effective PBIS implementation (Chitiyo and Wheeler, 2009), the present findings offer a nuanced perspective. Parents’ expressed motivation suggests that enhancing communication practices and creating more inclusive decision-making opportunities may help address these barriers.

### 5.4 Contribution to the literature

By focusing on parents’ lived experiences, this study adds a more personal and relational perspective to the existing body of research on PBIS implementation. Whereas much of the previous literature has concentrated on procedural fidelity and measurable student outcomes, the present findings highlight the importance of the relational connection between schools and families in shaping how PBIS is understood and experienced. Exploring how parents make sense of their involvement helps explain why partnership efforts may sometimes feel surface-level, even when formal systems for collaboration are established. The findings suggest that strengthening parental engagement in PBIS involves more than providing technical guidance. It also requires thoughtful communication, acknowledgment of parents’ contributions, and a genuine commitment to shared responsibility.

## 6 Limitations

This study has several limitations that should be acknowledged. First, the sample included only eight parents. While this number is appropriate for an Interpretative Phenomenological Analysis study, it limits the ability to draw broader conclusions about all families involved in PBIS. Second, the participants were recruited from schools located in two states, Pennsylvania and Maryland. Educational policies, school culture, and implementation practices may vary across regions, which may influence how parents experience PBIS. Additionally, the findings are based on parents’ self-reported experiences. These accounts reflect personal perceptions and may be influenced by memory, emotions, or individual interpretation. The study did not include perspectives from teachers or administrators, which may have provided a more comprehensive understanding of the collaboration process. Finally, all participating parents had children receiving Tier 3 PBIS support. Parents of students receiving Tier 1 or Tier 2 interventions may have different experiences that were not captured in this study. Although these limitations should be considered, the study still provides valuable insight into how parents experience and interpret their involvement in PBIS.

## 7 Summary, conclusion, and future research

The findings of this study suggest that parental involvement in PBIS is shaped not only by structural practices but also by how parents interpret their role within the school system. Although parents expressed strong motivation to participate, many experienced a limited understanding of PBIS processes and minimal inclusion in planning and decision-making. This indicates that engagement requires more than simply providing information; it also requires clear communication and shared responsibility between parents and schools. Previous research has emphasized that parents are critical partners in supporting positive behavioral outcomes (Chu, 2015; Garbacz et al., 2018). The present study adds to this literature by highlighting how parents experience their involvement at a relational level. When parents are informed but not meaningfully included, partnerships may remain procedural rather than collaborative. Strengthening collaboration between schools and families may empower parents to contribute more effectively to Behavior Intervention Plans and related supports (Minke and Anderson, 2005; Grüter et al., 2024). Structured communication, training opportunities, and shared planning discussions may help increase clarity and confidence among families. As suggested in previous research (Närhi et al., 2017), carefully planned and purposeful parental involvement may improve both behavioral and academic outcomes.

Future research should include larger and more diverse samples across different regions and school contexts to examine whether similar patterns are observed. Including perspectives from teachers and administrators may also provide a more comprehensive understanding of collaboration within PBIS frameworks. Additionally, studies exploring structured parent-training models within PBIS may help identify practical strategies for strengthening family and school partnerships. This study focused only on Tier 3 of PBIS. Future research could examine parental involvement across all PBIS tiers to provide a more complete understanding.

## Data Availability

The original contributions presented in this study are included in this article/supplementary materials, further inquiries can be directed to the corresponding author.
